# The Technology of the Gibbet

**DOI:** 10.1007/s10761-014-0275-0

**Published:** 2014-09-14

**Authors:** Sarah Tarlow

**Affiliations:** School of Archaeology and Ancient History, University of Leicester, Leicester, UK

**Keywords:** Body, Punishment, Criminal, Technology

## Abstract

The practice of “hanging in chains” or gibbeting had been part of the punitive repertoire of the English and Welsh judicial system for centuries before the 1751–52 Murder Act specified it as one of two mandatory post-mortem punishments for murderers. The practice was not abolished until 1834. This article considers the technical and design features of the gibbet cage, through an exhaustive survey and catalogue of their surviving remains. It notes that, given the comparative rarity of hanging in chains, no chronological or regional traditions of design are evident in this kind of artifact, since blacksmiths were individually solving the problem of fulfilling the necessary functions of a gibbet cage without knowledge of previous examples and under great time pressure. The technology of the gibbet shows how state directives intersected with geographical discretion in the creation of idiosyncratic local solutions.

Whereas the horrid crime of murder has of late been more frequently perpetrated than formerly…it is thereby become necessary, that some further terror and peculiar mark of infamy be added to the punishment of death

An Act for Better Preventing the Horrid Crime of Murder (Murder Act), 1751

## Introduction

In 1751, there were so many crimes carrying the death sentence that people began to worry that there was no way of distinguishing the most serious among them. To that end, a parliamentary act “for better preventing the horrid crime of murder” ordered that those convicted of murder should suffer some additional punishment. From the following year, when the Act became law, the bodies of those executed for murder could not be buried unless they had first been either given to anatomists for dissection, or “hung in chains.” Hanging in chains, also called gibbeting, involved placing the dead body inside a gibbet cage (an iron cage or framework) and suspending it from a high post. A new interdisciplinary project based at the University of Leicester and funded by the Wellcome Trust aims to examine how the body of the executed criminal was treated judicially, medically, scientifically, socially, and religiously during the key period between the Murder Act and the Anatomy Act of 1832. This latter piece of legislation aimed to regularize the supply of cadavers to anatomy schools in the wake of the Burke and Hare scandal and increasing anxiety about grave robbing (Richardson 1987). After 1832, executed criminals were generally buried within the prison precinct, and the needs of anatomists were supplied by the “unclaimed” bodies of the poor who died in workhouses or hospitals. After the summer of 1832 nobody was gibbeted in Britain, and the practice was formally abolished for England and Wales in 1834.

This paper considers the gibbet as an artifact, and in particular addresses some of the peculiarities of design evident in the corpus of surviving gibbet cages in England and Wales. Gibbets have never been described or catalogued before, and the literature on hanging in chains is predominantly non-academic. One of the project’s first tasks therefore was to undertake extensive research among primary historical sources and in the collections of local museums around the country. Project members Richard Ward and Zoe Dyndor constructed a database of capital convictions in England and Wales during the years between 1751 and 1832 with post mortem provision, based on historical sources such as the court sessions papers of the assize courts, sheriffs’ cravings (applications for reimbursement of expenses incurred by the sheriffs in managing the assizes and carrying out sentences) and newspaper records. According to these sources, the majority of murderers’ bodies (over 80 %) were sentenced to the anatomist’s slab. However, this paper concerns the 9.6 % who were “hung in chains” (around 6.5 % of convicted murderers were pardoned, a few were burned at the stake and around 2 % died in jail before their execution). Murderers made up about three quarters of those who were hung in chains, but the punishment was also ordered for other serious crimes, most frequently robbing the mail, piracy and smuggling.

## This Study

This article is particularly concerned with the technology of the gibbet and what that can tell us about craft traditions, design, and innovation; and about the interaction of state-level directive with local discretion. It makes use of a survey of all surviving gibbet cages in England and Wales, together with historical sources, notably newspapers; local histories, mostly nineteenth-century in date; and a number of documentary sources including most notably, the Sheriffs’ Cravings. Richard Ward of our research team has discovered a previously under-exploited source of evidence at the National Archives in Kew in the form of the expense claims submitted by sheriffs in relation to the costs of keeping remanded prisoners and executing sentences passed at the assize courts: the “Sheriffs’ Cravings” (TNA records beginning T90, Ward 2014).

## The Incidence of Hanging in Chains

**Fig. 1:**
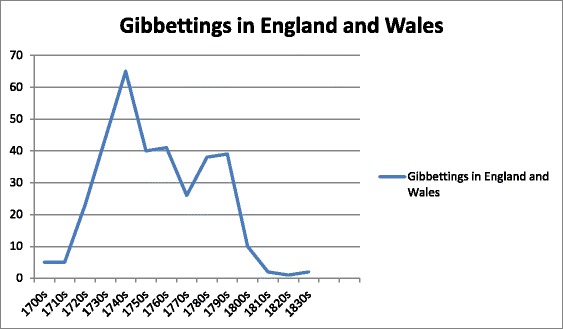
Incidence of gibbeting in England and Wales. 1700–1832

Hanging in chains had long been part of the repertoire of discretionary punishments a judge might choose to pronounce. In the seventeenth and eighteenth centuries, it was quite widespread practice simply to leave the body of the executed criminal hanging from the scaffold on which he was executed, which was often located at the scene of the crime (Poole 2008). Securing exact numbers of those hung in chains in the early part of the eighteenth century is not easy, but from the 1730s it has been possible to triangulate accounts of hanging in chains between newspaper sources, sheriffs’ cravings and other sources to arrive at a reasonably accurate figure. Therefore, the numbers in our graph probably undercount the number of gibbetings in the period 1700–30, although the general increase in numbers through the first half of the eighteenth century is real. Gibbeting of executed criminals peaked in the 1740s—before the passage of the Murder Act (Fig. 1).

By the end of the eighteenth century more than 220 offences carried the death penalty, including damaging the banks of canals and stealing from a rabbit warren. This compares to around 50 capital offences in 1688. The number of new capital offences created during the eighteenth century relate mostly to the protection of private property during the age of ascendant capitalism, and relate to anxieties of the owning classes about their personal security, the security of their property and the maintenance of public order during episodes of unrest (Hay 1975). The harshness of this legal regime has earned the period the soubriquet of the “Bloody Code” and, if one were looking only at the statutes, this period would seem the harshest in our judicial history. However, the actual number of death sentences passed by the assize court judges, who heard most such cases was far fewer than the number of convictions, which was fewer than the number of eligible offences that came to court, which was in turn smaller than the number of crimes committed (Gatrell 1994). Historical research has shown in fact that juries were often unwilling to convict in cases where the accused was likely to die, and prosecutors sometimes deliberately reduced the severity of the crime from a felony to a misdemeanor so that a lighter sentence might be obtained (King 2000, pp. 231–242). Project member Peter King notes also that in the north and west of England and in Wales an “unbloody code” could be said to prevail, in which convicts were extremely unlikely to be executed (King and Ward 2014). In England and Wales there were between four (in 1802) and 28 (in 1752) convictions for killing offences (murder, infanticide and petty treason) per annum between 1752 and 1832, averaging 14.5 a year. To these must be added those convicted of other offences and given a death sentence, most of whom would have had their bodies returned to their families for normal burial, unless some exceptional post-mortem punishment was given as part of the sentence or, as sometimes happened, the convict had sold their corpse privately for dissection.

Despite the law now insisting that the bodies of murderers be hung in chains or dissected, after the Murder Act the number of bodies hung in chains in fact declined. Altogether during the period 1752–1832 1,394 offenders were capitally convicted for killing offences in England and Wales, of whom 134 were hung in chains. There were also a number of property criminals, mostly convicted of robbing the mail, highway robbery, smuggling or piracy (considered by the Admiralty courts) who were given discretionary post-execution sentences of hanging in chains. This is actually a very small number and equates to fewer than two gibbetings a year across England and Wales, averaged through the period. Nearly all of them took place during the eighteenth century; there were only a handful of cases after 1800 (see Fig. 1). The two cases in 1832 date to the period after the passage of the Anatomy Act, which ended the practice of dissecting the bodies of the executed and probably represents a misunderstanding by a couple of judges who thought that they were now compelled to hang in chains for murder (in fact the alternative of burial within the prison precinct was the preferred option). The public outcry following these two gibbetings (William Jobling in Jarrow and James Cook in Leicester) effectively ended the practice that was formally abolished in 1834.

## Making the Gibbet

When a man (and all the gibbeted convicts in the records for this period were men; female bodies were always in demand by anatomists) was sentenced to hang in chains, it was the responsibility of the sheriff to make arrangements for the erection of a gibbet pole at a suitable location, and for the manufacture of a gibbet cage and whatever hooks, chains, or other tackle was necessary to suspend the cage. In addition a pulley or temporary scaffolding would be needed to hoist the heavy iron contraption into position and secure it. Gibbets were made for a single criminal and were not normally reused. A gibbeted criminal would be exhibited close to the scene of crime and could remain in his gibbet for many decades, so reuse was not practical.

Typically the body of a criminal was gibbeted within a day or two of being executed but sometimes there were longer intervals, especially when the body had to be transported some distance to the place appointed for gibbeting. Pirates for example were usually hanged at execution dock in London, but might then be transported many miles around the coast—to Devon or Norfolk say—to be gibbeted. Occasionally the judge recognized the time needed to prepare for a gibbeting. Thomas Nicholson, sentenced to execution and hanging in chains at Cumberland Assizes on August 22, 1767 had the date of his execution respited until August 31 in order to make the necessary preparations (Assize Calendar Cumberland, August 26, 1767). Even so, that gave only just over a week to have the gibbet irons made, a gibbet structure created and erected and a location prepared. Of the 38 cases for which the date of hanging in chains is explicitly stated in the records, 33 were gibbeted on the day of their execution. The other 5 executions took place between 1 and 4 days before gibbeting, and all except one were transported at least 26mi (42 km) from the place of execution to the place of gibbeting so the delay is probably caused by the need to transport the body to the site where the gibbet was erected. Where no separate date for gibbeting is given, as in the majority of cases, it is probable that gibbeting most frequently occurred on the day of execution.

**Fig. 2:**
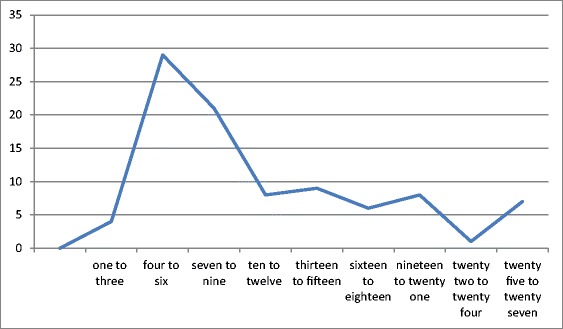
Interval between first day of assizes and date of execution; mean = 10.71

Since the date of conviction is not always known, we have calculated the interval between the first day of the assizes during which a criminal was convicted and the date of his execution. Of 101 cases in England outside London recorded in the Sheriff’s Cravings, the mean interval was 10.71 days, although there was considerable variation around this (Fig. 2). The Berkshire assizes at which Abraham Tull and William Hawkins were condemned began on the March 7, 1787 and they were executed and gibbeted on the 9th—only 2 days later, or even 1 day if their case was not heard on the first day of the assize sitting. Thomas Colley, on the other hand, was tried at the Hertfordshire assizes beginning on July 29, 1751, but not executed and gibbeted until the August 24, nearly a month later. Delays of more than 2 weeks, however, are uncommon. Given that the start of assizes is likely to be before the date of conviction in many cases (assize sittings could take up to a week in this period), we can assume that the smith would normally have a week or less to make a set of irons.

It was necessary therefore to start on the construction of a gibbet and a set of irons as soon as possible after a sentence had been passed. Where possible, the condemned man was measured for his set of irons before execution, an experience that could be quite horrifying for the condemned man. When Ralph Smith of Lincolnshire was being measured for his irons in 1792, for example, he found it impossible to retain the composure he had exhibited during sentencing, according to a contemporary newspaper report (*Lloyds Evening Post* 1792).

Even with the ability to start making the gibbet irons while the condemned man was still alive there could be considerable time pressure. Moreover, careful measuring of the body to be enclosed was not always possible, and sometimes the criminal resisted this horrible reminder of his imminent fate. Surviving gibbet cage structures show that they were often constructed so as to be adjustable to fit the size and shape of the particular body they came to enclose. On the Keal gibbet at Louth, for example, both the belt bands and the long straps are punched several times so that the framework could be extended or contracted and bolted into place to fit supportively close to the body. A similar design is evident on the leg iron of Tull or Hawkins’s gibbet in the collection of Reading Museum, which can be tightened to suit the circumference of the leg. While some gibbet cages, like James Cook’s of Leicester, have rigid, hinged hoops, others allow for some degree of shaping to the criminal’s body. Only a small part of Robert Matcham’s gibbet survives at Norris House Museum, St Ives: part of what is probably a waist belt, made of a series of five curved and hinged plates which would probably have conformed quite closely to the shape and size of the condemned man’s waist. The account of John Curtis’s hanging in chains in Wiltshire in 1764 mentions that the smith who made the chains was also responsible for fitting them and was paid to travel to the execution for this purpose.

**Fig. 3:**
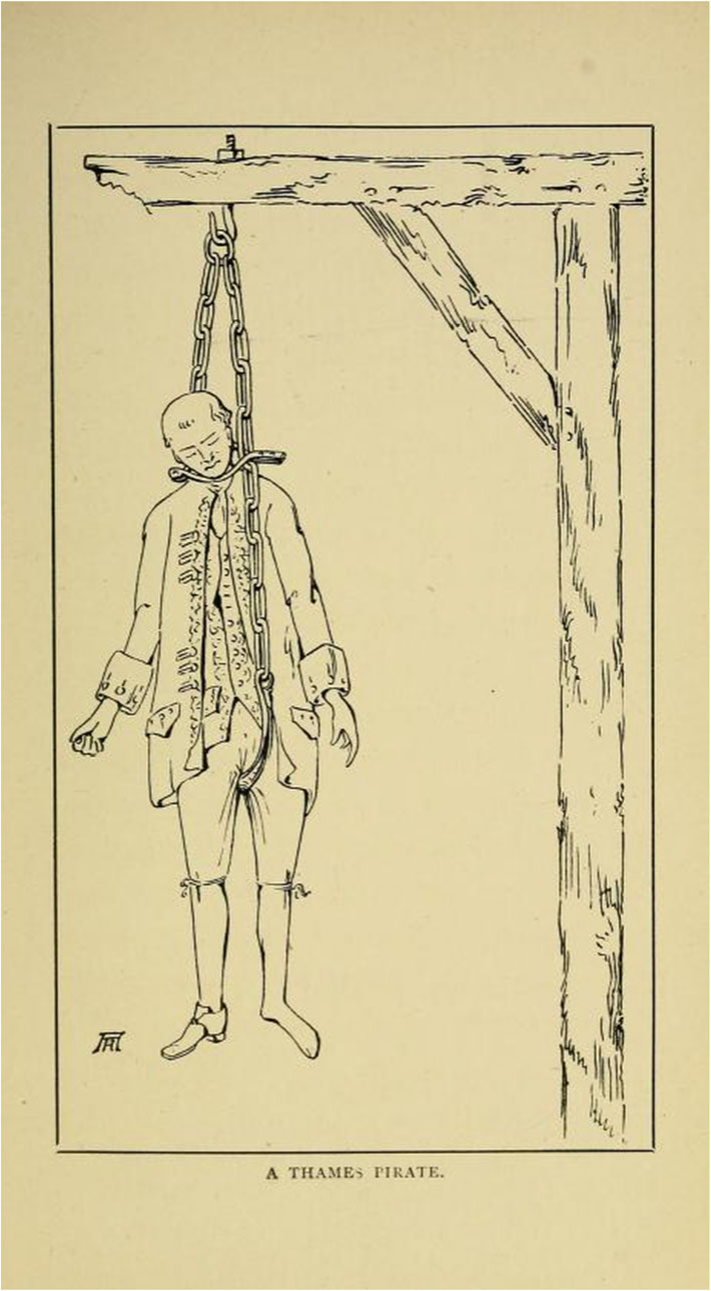
“A Thames Pirate” from Hartshorne 1893, p. 77. This single chain and neck brace would have done little to secure the body from theft

Despite attempts to make the gibbet irons adjustable, designs were not always successful: in 1750 the London Evening Post records that the body of John Barchard had to be taken back to the jail after his execution while the gibbet irons were altered, “they proving too little” (Sept. 29, 1750). Some gibbet contraptions were so basic that the size or shape of the corpse made little difference. Hartshorne (1893, p. 77) shows “a Thames pirate” suspended in what is apparently a single chain with a gusset passing between the legs and a brace around the neck to keep the body upright (Fig. 3). It would be easy to remove a body from such a rig, nor would it keep the body together for long once decay began to accelerate, so such a design could not have been a very successful gibbet.

Hanging in chains was an expensive business. Table 1 shows the claims submitted for the expenses incurred in the execution, transporting and hanging in chains of various criminals in the eighteenth century.

**Table 1:** Gibbeting expenses

Name	Year	Assize	Place of execution	Place of gibbeting	Distance from ex to gib	Date of Assize^a^	Date executed	Date gibbeted	Gibbeting expense	Cost of Irons	Total Execution Exp
John Sturabout	1738	Berkshire	Tilehouse Heath	Tilehouse Heath	0	27/02/1738	11/03/1738	11/03/1738	£13, 17s	£7, 7s	£24, 7S
Thomas Watkins	1764	Berkshire	Market Place, Windsor	Gallows Lane, Windsor	minimal	5 /03/1764	9/03/1764	9/03/1764	Not Specified	Not Specified	£26, 5s
Abraham Tull and William Hawkins	1787	Berkshire	Mortimer Common	Mortimer Common	0	7/03/1787	9/03/1787	9/03/1787	Not Specified	Not Specified	£60, 8s, 9
Thomas Marsh and Richard Marshall	1736	Bucks	Wycombe	Wycombe	0	8/03/1736	22/03/1736	22/03/1736	£10, 10s	Not Specified	£21
George Davies	1755	Bucks	Gerrards Cross - Holtspur Heath, road leading from Beaconsfield to High Wycombe	Gerrards Cross - Holtspur Heath, road leading from Beaconsfield to High Wycombe	0	10/03/1755	31/03/1755	31/03/1755	£10, 10s	Not Specified	£23, 2s
Edward Corbett	1773	Bucks	Buckingham	Bierton	4 miles	19/07/1773	23/07/1773		£12, 12s	Not Specified	£17, 17s
Samuel Thorley	1777	Cheshire	Boughton - Chester	Congleton Heath	30 miles	3/04/1777	10/04/1777	11/04/1777	£25	Not Specified	£25
John Dean	1790	Cheshire	Boughton - Chester	Stockport Moor	42 miles	31/08/1790	2/09/1790		£56, 12s	£17, 18s	£84, 8s, 3
Henry Clark	1791	Cheshire	Boughton - Chester	Helsby Tor	8 miles	14/04/1791	21/04/1791		Not Specified	Not Specified	£68, 1s, 7
John Price and Thomas Brown	1796	Cheshire	Boughton - Chester	Trafford Green	9 miles	4/04/1796	30/04/1796		Not Specified	Not Specified	£105, 19, 9.5p
Thomas Nicholson	1767	Cumbria	Penrith	Penrith	0	25/08/1767	31/08/1767	31/08/1767	£15, 15s	Not Specified	£60, 5s, 11.5
John Whitfield	1769	Cumbria	Carlisle	Armathwaite	13 miles	29/07/1769	9/08/1769	9/08/1769	£28, 15s	Not Specified	£28, 15s
Thomas Dyer	1746	Devon	Woodbury Down	Woodbury Down	0	17/03/1746	11/04/1746	11/04/1746	£8, 8s	£4, 4Ss	£8, 8s
John Young	1752	Devon	Exeter	45 miles from Exeter	45 miles	16/04/1769	3/04/1752		Not Specified	Not Specified	£40
John Andrews	1781	Devon	Exeter	unknown	unknown	6/08/1781	24/08/1781		Not Specified	Not Specified	£15, 15s, 6
Francis Martin	1793	Devon	Haldown	Haldown	0	18/03/1793	28/03/1793	28/03/1793	Not Specified	Not Specified	£30
Thomas Campion	1795	Devon	Bovey Heath	Bovey Heath	0	27/07/1795	6/08/1795	6/08/1795	Not Specified	Not Specified	£23, 1s, 6
John Williams and Richard Smith	1788	Devon	Exeter	Stoke	42 miles	17/03/1788	24/03/1788	28/03/1788	£18, 18s	£10, 10s	£51, 1s
Robert Rymes	1767	Dorset	Western Road	Western road	0	19/03/1767	24/03/1767	24/03/1767	£5, 10, 7	Not Specified	£9, 15s, 7
John Swan	1752	Essex	Epping forrest	6 miles from exec	6 miles	9/03/1752	28/03/1752	30/03/1752	Not Specified	Not Specified	£56
Matthew Snatt	1757	Essex	Chelmsford	Epping Forrest	2 miles	29/07/1757	12/08/1757		Not Specified	Not Specified	£9, 9s
James pookey	1758	Essex	Chelmsord	Walthamstow	25 miles	13/03/1758	18/03/1758		£13	£4	£19
Benjamin downing	1759	Essex	Chelmsford	Radwinter	25 miles	12/03/1759	30/03/1759		£7, 1s, 6p	£3, 10s	£14, 8, 6
William Saville	1790	Essex	Manuden	Manuden	0	8/03/1790	15/03/1790	15/03/1790	Not Specified	Not Specified	£8, 5s
Edmund Goodrich	1735	Gloucestershire	Gloucester	Corse Lawn, Cheltenham	5 miles	9/08/1735	22/03/1735		Not Specified	Not Specified	£40
Joseph Abseney	1749	Gloucestershire	Durdham Down	Durdham Down	0	5/08/1749	25/08/1749	25/08/1749	Not Specified	Not Specified	£30
Thomas Hanks	1763	Gloucestershire	Gloucester	Wick Bissington	26 miles	9/03/1763	14/03/1763	16/03/1763	£12, 12s	Not Specified	£22, 1s
William Keeley	1772	Gloucestershire	Campden	Campden	0	22/08/1772	28/08/1772	28/08/1772	Not Specified	Not Specified	£15, 15s
Thomas and Henry Dunsden	1784	Gloucestershire	Capslodge Plain, Wynchwood	Capslodge Plain, Wynchwood	0	24/07/1784	30/07/1784	30/07/1784	Not Specified	Not Specified	£30
Michael Moorey	1737	Hampshire	Winchester	Arreton, IoW	30 miles	2/03/1737	19/03/1737		£9, 15s, 6	Not Specified	£11, 15s, 6
Francis Arsine	1761	Hampshire	Winchester	Blockhouse Point, Portsmouth	30 miles	29/06/1761	4/06/1761	4/06/1761	£12, 8s	Not Specified	£20, 6
John Bryan	1781	Hampshire	Winchester	Blockhouse Point, Portsmouth	30 miles	24/07/1781	30/07/1781		Not Specified	Not Specified	£5
Francis Jennison and William Butterworth	1794	Hampshire	Langton Harbour	Cumberland Fort	0	29/07/1794	04/07/1794	04/07/1794	Not Specified	Not Specified	£9, 4s
William Spiggott and William Evan	1770	Herefordshire	Hereford	Hardwick Common, Hay	16 miles	24/07/1770	30/07/1770		£21	Not Specified	£33, 12s
William Jones	1790	Herefordshire	Hereford	Longtown Green	25 miles	29/07/1790	2/08/1790		Not Specified	Not Specified	£25, 5s, 1
Thomas Colley	1751	Hertfordshire	Gubblecut	Gubblecut	0	29/07/1751	24/08/1751	24/08/1751	Not Specified	£5, 0, 0	£26, 7, 5
John Gatward	1757	Hertfordshire	Colliers End	Colliers End	0	11/04/1757	27/04/1757	27/04/1757	£10, 0, 0	£5, 0, 0	£20, 10, 0
Daniel Ginger	1761	Hertfordshire	Colney	Colney	0	4/03/1761	11/03/1761		£10, 5s	Not Specified	£22, 17s
Gervaise Matcham	1787	Huntingdon	Huntingdon	Wooly Road Junction	3 miles	29/07/1787	2/08/1787		£10, 10s	Not Specified	£10, 10s
David Anderson	1736	Kent	Hambledown	Hambledown	0	16/08/1736	2/09/1736		£16, 6s	£6, 6s	£26, 6s
William Fairall	1749	Kent	Tyburn	Horsemonden	over 50 miles	5/04/1749	26/04/1749		£13	£6	£24, 1
Thomas Kingsmill	1749	Kent	Tyburn	Goudhurst Gore	over 50 miles	5/04/1749	26/04/1749		£13	£6	£24, 1
John Grace	1752	Kent	Hoo Common	Hoo Common	0	29/07/1752	13/08/1752	13/08/1752	£13	£6	£19
William Moore	1758	Kent		Chatham		20/03/1758	27/03/1758	29/03/1758	£4	Not Specified	£4
Jean Baptiste Picard	1761	Kent	Sisinghurst?	Sisinghurst?	0	16/03/1761	25/03/1761		£2, 10s	Not Specified	£8
John Knight	1780	Kent	Maidstone	Whitstable	30 miles	13/03/1780	18/03/1780		£6, 10s	Not Specified	£19, 10s
Charles Storey	1782	Kent	Maidstone	Cartham	30 miles	22/07/1782	26/07/1782		Not Specified	Not Specified	£12, 12s
John Grindrod	1759	Lancashire	Lancaster	Pendleton Moor	45 miles	17/03/1759	24/03/1759		£8, 3s	Not Specified	£18, 1s
Edward Miles	1793	Lancashire	Lancaster	Warrington	55 miles	10/08/1793	14/09/1793		£49, 17s, 4.5p	Not Specified	£67, 8s, 6.5
Thomas Brown	1759	Lincolnshire	Ancholme Corner	Ancholme Corner	0	12/03/1759	28/03/1759	28/03/1759	£12, 5s	Not Specified	£17, 10s
William Williams	1731	Middlesex	Tyburn	Turnham Green		24/02/1731	8/03/1731		£10, 9s	£6, 5s	£19, 11s
John Field, Joseph Rose, William Saunders and Humphrey Walker	1735	Middlesex	Tyburn	Edgware		26/02/1735	10/03/1735		£41, 10s	£25	£48, 17s
Samuel Gregory	1735	Middlesex	Tyburn	Edgware Road		26/03/1735	4/07/1735		£14, 10s	£6, 5s	£19, 15s
William Blackwell	1735	Middlesex	Tyburn	near Paddington		15/10/1735	10/11/1735		£12	£5, 5s	£17, 5s
William Maw and Jeffrey Morat	1737	Middlesex	Tyburn	Shepherds Bush		16/02/1737	3/03/1737		£19, 2s	£10, 10	£28, 11s
James Cauldclough and Joseph Morris	1739	Middlesex	Tyburn	Hounslow Heath		7/06/1739	2/07/1739		£21, 2s	£12, 12s	£30, 11s
John Shilling	1786	Norfolk	Norwich	Badley Moor	16 miles	17/03/1786	25/03/1786		£32, 17s	Not Specified	£32, 17s
William Anthony	1792	Norfolk	Norwich	Kettlestone Common	24 miles	16/03/1792	24/03/1792		£10	Not Specified	£10
Stephen Watson	1795	Norfolk	Thetford	West Bradenham	20 miles	20/03/1795	25/03/1795		£14	Not Specified	£29
William Bennington	1795	Norfolk	Thetford	Dereham	20 miles	20/03/1795	25/03/1795		£14	Not Specified	£29
William Suffolk	1795	Norfolk	Norwich	North Walsham	15 miles	17/03/1797	24/03/1795		£24, 8s, 4p	£7, 8s, 5p	£39, 16, 9s
John Croxford	1764	Northants	Northampton	Hollowell Green	10 miles	2/08/1764	4/08/1764		£30	Not Specified	£30
Michael Curry	1739	Northumbers	Newcastle	Hartley	10 miles	20/08/1739	4/09/1739		Not Specified	Not Specified	£20
William Winter	1792	Northumbers	Newcastle	Steng Cross	35 miles	4/08/1792	10/08/1792		Not Specified	Not Specified	£55
Robert Downe	1767	Notts	Nottingham	Mansfield Forest	1 mile	8/08/1767	10/08/1767		£5, 5s	Not Specified	£10, 10
John Spencer	1779	Notts	Nottingham	Scrooby	30 miles	22/07/1779	26/07/1779		Not Specified	Not Specified	£8, 8s
John Bowland	1769	Rutland	Oakham	Empingham common	6 miles	7/07/1769	20/7/1769		£11, 18s	£5, 15s, 6p	£25, 3s
Thomas and Joseph Darby	1759	Shropshire	Salisbury	Oldbury	40 miles	6/08/1759	11/08/1759		£16, 5s	£10, 10s	£40, 8s, 10
John Scott	1767	Shropshire	Bridgnorth	Bridgnorth	0	4/04/1767	21/04/1767	21/04/1767	£16, 16s	Not Specified	£32, 11, 6
Thomas Limpous	1739	Somerset	Dunkit Hill	Dunkit Hill	0	29/08/1739	21/09/1739	21/09/1739	£10, 17s, 9p	£5, 5s	£32, 1s, 6
Francis Wilkins	1746	Somerset	Taunton	Black Down	5 miles	2/09/1746	unknown		£9, 9s	£5, 5s	£14, 9s
John Galaway	1746	Somerset	Taunton	Chilton Heath	6 miles	2/09/1746	unknown		£9, 9s	£5, 5s	£14, 9s
Richard Williams	1799	Somerset	Ilton	Ilton	0	28/03/1799	1/04/1799		£10	Not Specified	£10
Thomas Willott	1749	Staffordshire	Mere Heath	Mere Heath	0	15/03/1749	unknown		Not Specified	Not Specified	£35, 10s, 6
George Easthop	1779	Staffordshire	Bradbury Heath	Bradbury Heath	0	25/03/1779	29/03/1779	29/03/1779	Not Specified	Not Specified	£25
Thomas Otley	1752	Suffolk	Bury St Edmonds	Black Close Hill		23/07/1752	27/07/1752		£16, 12s, 2d	£10, 15s, 6p	£16, 12s, 2d
Edward Johnson	1761	Suffolk	Sudbury	Sudbury	0	16/03/1761	23/03/1761	23/03/1761	£4, 6s, 6p	Not Specified	£9, 11s, 6
Roger Benstead	1792	Suffolk	Bury St Edmonds	Undley Common	17 miles	21/03/1792	26/03/1792		£10, 11s, 6p	£5, 17s	£27, 7s
John Nichols	1794	Suffolk	Bury St Edmonds	Honington	10 miles	19/03/1794	26/03/1794		£11, 4s, 6p	£6, 10s	£25, 5s
John James and Joseph Emerson	1735	Surrey	Kennington Common	Kennington Common	0	6/08/1735	20/08/1735		£20	Not Specified	£20
Gill Smith	1738	Surrey	Kennington Common	Kennington Common	0	16/03/1738	10/04/1738	10/04/1738	£12, 12s	Not Specified	£15, 12s
William Corbett	1764	Surrey	Kennington Common	Rotherhithe	4 miles	29/03/1764	6/04/1764		£10	Not Specified	£15
Lanigan, Marshall and Casey	1787	Surrey	Hindhead	Hindhead	0	2/04/1787	7/04/1787		Not Specified	Not Specified	£53, 8s, 2
John Mills	1749	Sussex	Slindon Common	Slindon Common	0	13/03/1749	20/03/1749	20/03/1749	£10, 18s, 9p	£9, 9s with Sheerman	£10, 18s, 9
Henry Sheerman	1749	Sussex	Rake	Rake	0	13/03/1749	21/03/1749	21/03/1749	£10	£9, 9s with Sheerman	£10
Edmund Richards	1749	Sussex	Hambrook Common	Hambrook Common	0	29/07/1749	9/08/1749	9/08/1749	£3, 8s, 9p	£9 for Richards and Chapman	£3, 8s, 9
George Chapman	1749	Sussex	Hurst Common	Hurst Common	0	29/07/1749	19/08/1749	19/08/1749	£14, 15s, 6p	£9 for Richards and Chapman	£14, 15s, 6
John Upperton	1771	Sussex	Horsham	Wepham Wood	25 miles	18/03/1771	6/04/1771		£5, 5s	Not Specified	£12, 12s
Edward Howell and James Rook	1793	Sussex	Horsham	Shoreham	25 miles	18/03/1793	23/04/1793		£20	£10	£30, 10s
Robert and William Drewitt	1799	Sussex	Horsham	North Heath Common, Midhurst	40 miles	25/03/1799	13/04/1799		£35, 8s	£10, 10s	£45, 18s
Thomas Savage	1737	Warwickshire	12 miles from gaol	12 miles from gaol	0		unknown		£7, 7s	Not Specified	£21, 1s, 10
Edward Drury, Robert Lesley, Moses Baker	1765	Warwickshire	Stoneleigh Common	Stoneleigh Common	0	6/04/1765	17/04/1765	17/04/1465	£45	£25	£55, 10
Thomas Hammond and John Pitmore	1781	Warwickshire	Washwood Heath	Washwood Heath	0	27/03/1781	2/04/1781	2/04/1781	£47, 6s, 2p	Not Specified	£52, 2s, 2
John Clay	1783	Warwickshire	Warwick	Chilvers Coton	16 miles	22/03/1783	29/03/1781		Not Specified	Not Specified	£2, 16s
William Jaques	1764	Wiltshire	Stanton Fields	Stanton Fields	0	4/08/1764	14/08/1764	14/08/1764	£9, 10s	£5	£18
John Curtis	1768	Wiltshire	Hernham Hill	Hernham Hill	0	5/03/1768	14/03/1768		£9, 8s, 1p	£3, 10	£14, 13s, 1
John Franklin	1770	Wiltshire	Bockington Abney	Bockington Abney	0	31/03/1770	20/04/1770		Not Specified	£5, 5s	£17, 5s
William Buckley	1762	Worcestershire	Worcester	Wyre Forrest	15 miles	11/08/1762	14/08/1762		£7	£5, 5s	£8, 1s
Eugene Aram	1759	Yorkshire	York	Knaresborough Forest	18 Miles	28/07/1759	6/08/1759		Not Specified	Not Specified	£12, 9s
Thomas Lee	1768	Yorkshire	York	Grassington Gate	40 miles	16/07/1768	25/07/1768		Not Specified	Not Specified	£20
Robert Thomas	1774	Yorkshire	York	Beacon Hill, Halifax	80 miles	16/07/1774	6/08/1774		Not Specified	Not Specified	£90
Matthew Norminton	1776	Yorkshire	York	Beacon Hill, Halifax	80 miles	9/03/1776	15/04/1776		Not Specified	Not Specified	£40
Spence Broughton	1792	Yorkshire	York	Attercliffe	50 miles	19/03/1792	14/04/1792		£39, 15s, 2p	Not Specified	£71, 10, 2

^a^Start Date of Assize, not necessarily date of conviction

## From the Gallows to the Gibbet

In the eighteenth and nineteenth centuries in Britain, bodies were always dead before being placed into gibbet cages (although in earlier centuries and in other parts of the world criminals were occasionally gibbeted alive and left to die of starvation and exposure. This was a well-known punishment for rebellious slaves in the Caribbean during this period (see Sheridan 1974, p. 265). Once signs of life were no longer evident, the body would be removed from the scaffold and prepared for gibbeting. Several secondary sources suggest that it was normal to coat the body with tar before gibbeting it, although there is little clarity about what this meant. Despite a thorough search, it has been impossible to find in the primary sources any detail of this practice, and it is rarely mentioned except in some later newspaper accounts. None of the sheriffs’ cravings ever include tar or anything like it as an itemized expense, even when other apparently trivial costs such as a stool or some ale for the wagoners, are separately noted. If “tar” was used routinely it was probably only a small quantity of something like creosote, the application of which would not prevent recognition of the criminal, take much time or impede the process of enclosing the body in its gibbet cage. The body would then be fitted into its gibbet cage and transported by cart to the place of display. Usually the fitting of the irons is not described in the sources, but occasionally the cravings mention a cost for having the smith attend the execution in order to fit the irons afterwards. When the novelist Rider Haggard discovered the remains of the gibbet and skeleton of Stephen Walton while digging on West Bradenham common, Norfolk, he noticed that the skull had clear scorch marks where it had been burned by a hot iron, thus proving to Rider Haggard that the man must have been dead when enclosed in his gibbet cage, and to us that the smith on that occasion fitted the gibbet by soldering or welding (Rider Haggard 1899, p. 355). The unusual return of John Barchard’s body to the jail so that the irons could be resized suggests that normally the irons were fitted directly after and at the scene of execution. This also constitutes circumstantial evidence that tarring the body was not normally practised, or was a very quick and easy process, since it is hard to see how a corpse could be stripped, immersed in tar, redressed and fitted into irons in a very short time and at the foot of the scaffold.

The body in its chains was then taken by cart to the place where the gibbet had been erected. This location was normally specified in the sentence. Usually the places chosen were as close as practical to the scene of the crime but also visible from the public road. Because of the large crowds attracted, open locations away from densely populated areas were also generally preferred (Tarlow and Dyndor 2014). The poles from which the cages were hung were often very high (10 m or more), which discouraged attempts to rescue the body or to steal the gibbet, and chains which comprised a substantial quantity of iron. The post was also sometimes fitted with spikes around the bottom to make it hard to scale. The gibbet post of Adam Graham, executed in 1748 and hung in chains on Kingmoor, Carlisle, was apparently 12yds (11 m) high and had 12,000 nails in it to prevent it being scaled or cut down to remove the body (Hartshorne 1893, pp. 66–67). The sheriffs’ cravings for Hampshire in 1761 note that when Francis Arsine was hung in chains the gibbet was “20 ft high made of very strong timber and secured with nails to prevent its being cut down and [fitted with] a secure set of chains.”

Several London gibbeting accounts (in the sheriffs’ cravings) make reference to “plating the gibbet.” The fairly detailed accounts for the gibbeting of Thomas Willot in Staffordshire in 1739 include “timber for the gibbet 28 ft long (being 7 yards or thereabout above ground) and cross pieces and carriage there of workmanship of the timber and erecting the gibbet and lining gibbet on each side with bars of iron.” Similarly, the cravings account for the gibbet of William Corbett (executed in Surrey in 1764) itemizes “the gibbet made strong with iron to prevent it being cut.”

While normally a special gibbet post would be prepared and erected, there are also cases where a tree was apparently used, although the risk of bodies or gibbets being removed from a tree would presumably be greater than from an isolated post, trees being generally more scalable. The gibbets of Tom and Harry Dunsdon, notorious highway robbers, murderers and general bruisers of Gloucestershire, were described as having been hung from a tree on the edge of Wychwood forest in 1784 (Darby 2011, p. 23), but the sheriffs’ cravings for the event record payments for wood and the services of a carpenter so it is probable that the word ‘tree’ referred here to the gibbet structure. When William Williams was gibbeted on Hounslow Heath in 1734 a sum of money was paid for “plating the tree,” the word “tree” being used in the sense of “scaffold” or alluding to part of the wooden structure: an item from the Worcestershire cravings of 1762 mentions the cost of “a tree [for a gibbet] 35 ft high and to the carpenter for making the same.”

The gibbet pole seems almost always to have been made from timber, although sometimes the cravings specify that the timber is “strong” or note that nails or iron bars or plates, as discussed above, should reinforce the main post. The sheriff who commissioned the three armed gibbet erected for the bodies of Drury, Barker and Lesley in Warwickshire in 1765 lists “materials of stone and timber” for the gibbet, but stone is not usually mentioned in connection with a gibbet, and there is no indication of what its role was to be—perhaps to construct a strong socket for the post. A broken socket stone at Gonerby Hill Foot, Lincolnshire, is believed locally to have supported a gibbet at one time (http://www.lincstothepast.com/photograph/ 290331.record?pt=S).

## At the Scene of the Gibbet

The newly gibbeted body was a great public attraction. Given the infrequency of hanging in chains in most areas, crowds of sometimes many thousands visited the gibbet in the days following its erection. George Drabble, the landlord of the Arrow pub close to the rough ground between Sheffield and Rotherham on which Spence Broughton was gibbeted in 1792 claimed to have made his fortune from the crowds who came to view his body, an estimated 40,000 on the first day alone (Knipe 1867, pp. 125–126). The gibbeting of Tom Otter in Lincolnshire in 1806 “inaugurated a week of merry-making of the most unseemly character. Booths were pitched near the gibbet, and great numbers of the people came to see the wretch suspended” (Andrews 1899, p. 68).

**Fig. 4:**
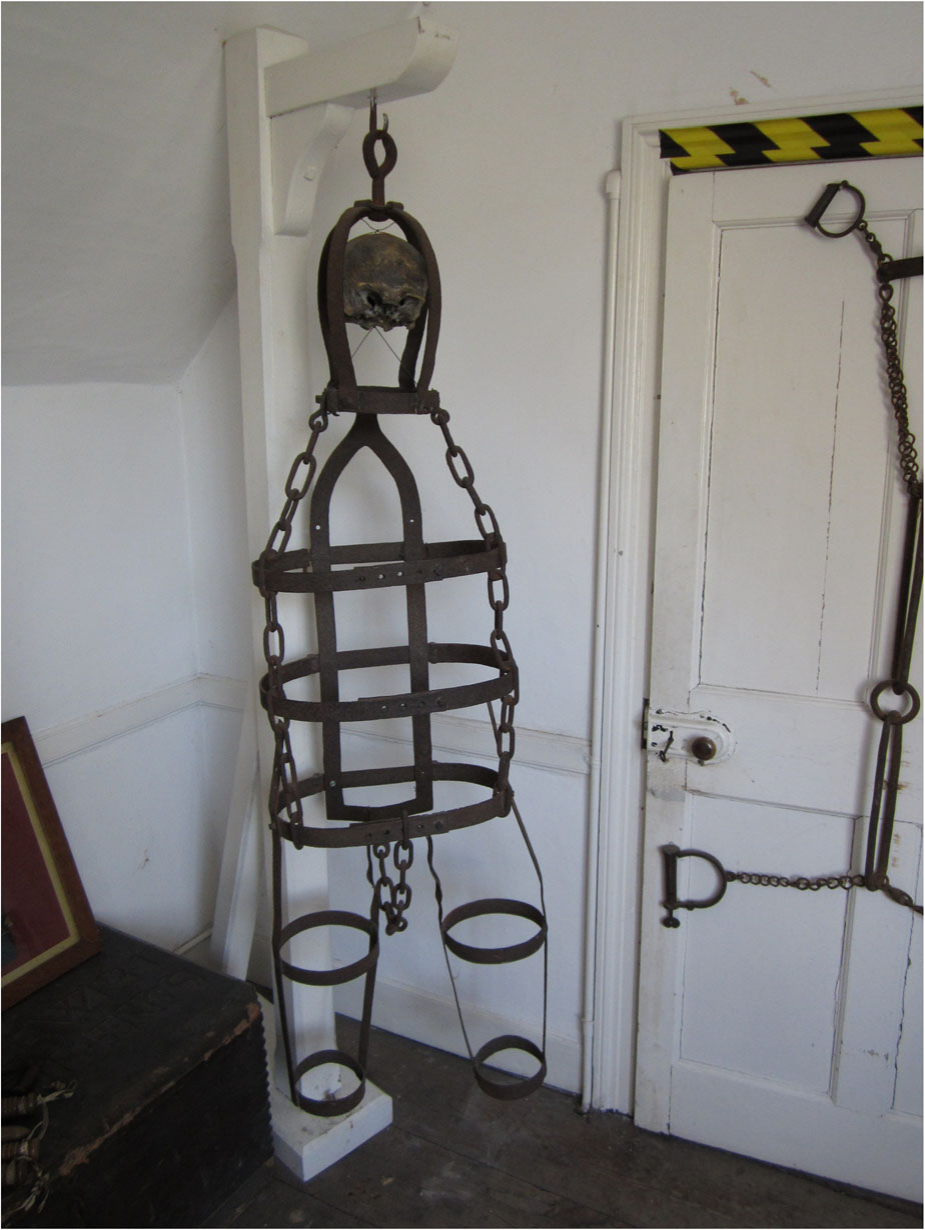
The gibbet cage of John Breeds, Rye, with parts of his skull still inside

The gibbet, complete with remains, might stay standing for many decades. Spence Broughton’s gibbet, mentioned above, stood from 1792 until 1827 or 1828 (Knipe 1867, p. 125). When it eventually did come down the gibbet cage and any bones that had not already fallen out suffered a range of fates. Sometimes the remains might be buried—as was reportedly the case with Anthony Lingard in Derbyshire and William Jobling in Jarrow (Andrews 1899, pp. 71, 74). The gibbet might be buried with the remains still inside, as happened in the cases of John Breeds in Rye (Fig. 4) and James Cook in Leicester, or retained as a curio for a private collection or public museum. Edwin Jarvis, who collected part of the gibbet of Tom Otter when it blew down in a storm in 1850, noted that gypsies had taken most of the metal. Unreliable sources also claim that enterprising recyclers apparently also got hold of Anthony Lingard’s gibbet cage and made it into toasting forks for sale to memento hunters (http://www.geograph.org.uk/photo/1564301), and metal from Black Toby’s gibbet from Suffolk was made into a thatching comb (Wensley Wright 1964, p. 187). While the toasting forks and thatching combs were probably just rather ghoulish souvenirs, and useful household items, other gibbets, especially in earlier periods had stranger powers. The gibbet of Andrew Mills, hung in chains in 1683 for murder at a site in County Durham that became known as “Andrew Mills’s stob” was, according to Andrews (1899, p. 46) “taken away bit by bit as it was regarded a charm for curing toothache.”

## Extant Gibbets

I have only been able to discover the whereabouts of 16 extant gibbet cages or partial cages, despite a thorough literature and online search and an appeal on national radio. The majority of gibbets seem to have disappeared. Their variable fates are considered below. Those gibbets that do exist are in a variety of styles. The surviving evidence is considered in Table 2.

**Table 2:** Extant gibbet cages or partial cages

Date	Name	County	Present location
?1720	Siôn Y Gof	Powys	St Fagan’s Museum of Welsh Life
1731	Keal	Lincs	Louth Museum
1742	Breeds	Sussex	Rye Town Hall
? late C18	Anon	London	Museum of London Docklands
?1777	?Hill	Hampshire	Winchester Westgate
1785	Cliffen	Norfolk	Norwich Castle Museum
1786	Matcham	Huntingdonshire	St Ives Museum
1787	Tull or Hawkins	Berkshire	Reading Museum
1791	Miles	Lancs	Warrington Museum
1792	Broughton?	S. Yorks	Weston Park Museum Sheffield
1794	Nicholls	Suffolk	Moyses Hall Museum, Bury St Edmunds
1795	Watson	Norfolk	Norwich Castle Museum
1795	Quin or Culley	Cambs	Wisbech and Fenland Museum
1806	Otter (Temporell)	Lincs	Doddington Hall
1832	Jobling	Nurthumberland	South Shields Museum (replica?)
1832	Cook	Leics	Nottingham Galleries of Justice (replica in Leicester Guildhall)

### Siôn Jones (Siôn Y Gof), (Dylife, Powys)

Siôn y Gof (Sion the Smith) was a blacksmith at Dylife, the site of a large lead mine. Convicted of murdering his wife and children and concealing their bodies in a disused mine shaft, he was one of a very small number of men sentenced to be hung in chains in Wales. The headpiece of his gibbet cage, still containing his skull was uncovered in 1938 and it is now in St Fagan’s museum of Welsh life. It was photographed and described by Iorwerth Peate (1939). It consists of a headpiece with three vertical straps attached to a collar. Although the lower part of the gibbet cage is missing, the straps plainly continue below the collar and were formerly attached to something, despite claims in the popular literature that Siôn y Gof’s head was hung in a separate gibbet to the rest of his body (e.g., Sale 2006, p. 41). Popular tradition also claims that Siôn Y Gof had to make his own irons (http://www.mysteriousbritain.co.uk/wales/powys/ hauntings/dylife-lead-mine.html-1), but this is unsubstantiated.

### John Keal (1731)

**Fig. 5:**
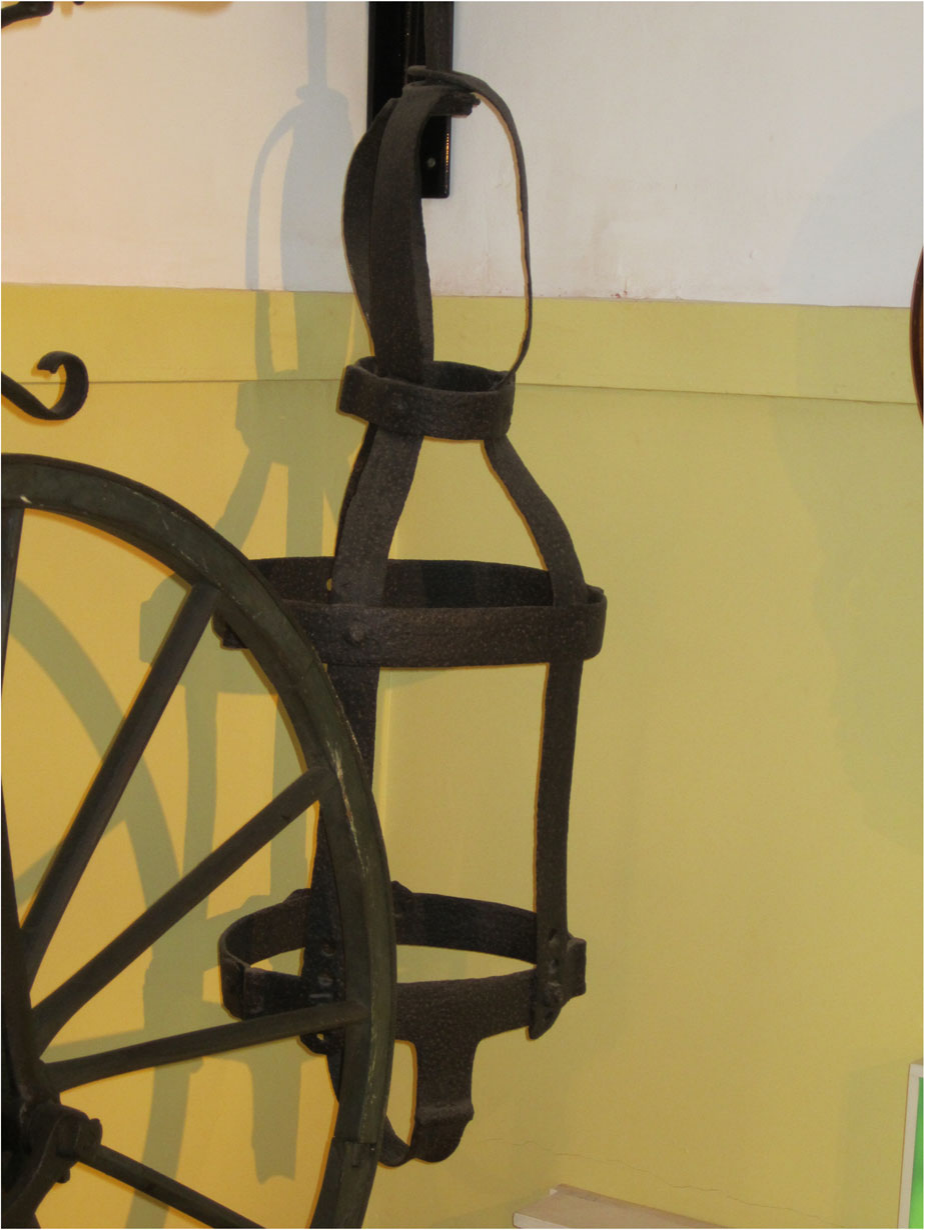
John Keal’s gibbet cage, Louth

John Keal was executed for the murder of his wife and child and gibbetted somewhere in Louth, Lincolnshire, on March 18, 11 days after his conviction at the Lincoln spring assizes, although there is some confusion about the exact location of the gibbet (McNeaney 2003, p. 70). After his body was taken down the post was used first in the stables of Louth’s House of Correction and then when that was demolished, the prison governor had the post turned into souvenirs and mementoes. The gibbet cage, however, is still remarkably complete and is on display in Louth museum (Fig. 5). It takes the form of three vertical strips of iron joined at the top, fastened to three circular bands around the neck waist and hips. The lowest band is attached to a hinged gusset. This provides a stable but basic framework for containing a corpse, although only the head and torso are supported; the arms and legs would hang outside the framework unsupported. The bands and belts have punched holes and are adjustable to fit the size of the body.

### John Breeds (Rye, 1742)

John Breeds was hung in chains in 1742 for the murder of the Deputy Mayor. After many years of exposure on Gibbet Marsh the body of John Breeds mostly disappeared into the marsh. The cage was retrieved by the Town Corporation and is now stored at Rye town hall. It still contains part of Breeds’s skull.

The head is enclosed by four curved vertical straps connected at the bottom by a neck brace. The main part has solid straps at the back and three hoops around the torso. The hoops are connected by links of iron chain, and a further length of chain forms the gusset. Each leg piece consists of an iron strap at the outside and at the inside of each leg with two hoops holding them together. There are no separate arm pieces but the torso bands are wide and so the arms probably went inside. The hook on the top of the headpiece shows considerable wear.

### Unknown, Museum of London Docklands

The Museum of London Docklands holds a gibbet cage of unknown provenance. The museum catalogue assigns it to the second half of the eighteenth century and relates it to the victims of the Admiralty Courts who were hanged at Execution Dock and gibbeted along the Thames estuary. This cage has a headpiece, a collar, and a gusset suspended from the collar and holding two iron hoops around the torso. Long iron bars run along the sides of the torso down the outside of the legs. Smaller iron hoops hold the legs in position and the feet are supported by stirrups. There are no arm fittings so presumably the arms would have hung loose outside the cage. The torso hoops and the collar are hinged and bolted, but the leg hoops cannot be opened and are not adjustable. The swivel eye above the headpiece shows wear. The history of this gibbet is not known, but it has at least two different episodes of workmanship—the two middle hoops of the left leg and the right vertical bar are of a different finish to the rest of the piece. This suggests that the gibbet cage was repaired or maintained, and it might be that it was reused. If it is a gibbet from the Thames estuary, used for those condemned by the Admiralty Court, it would have had a different tradition of use to the other gibbets described here—located by tradition rather than by scene of crime, and possibly re-used.

### James Cliffen (Dereham, Norfolk, 1785)

Only the headpiece of the gibbet cage of James Cliffen survives and is on show in Norwich Castle Museum. It is a substantial iron artifact of two bars crossed and bent to form a cage with four vertical bars. One of these is cut short, presumably to allow the face to show properly; the others are attached to a hinged collar. The swivel eye on top is nearly worn through.

### Gervase Matcham (Huntingdonshire, 1786)

Gervase Matcham was executed in 1786 for murder and gibbeted adjacent to the Great North Road (now the A1) in Huntingdonshire. A sketch map of his gibbet site was made in the 1920s when a low mound was still observable there. The site is now inaccessible because it is within the campus of Huntingdon Life Sciences, but maps and part of the gibbet cage are kept at the Norris museum, St Ives, Cambridgeshire. The only surviving part of the irons is an iron belt of five curved and hinged plates, with a keyhole shape punched through one end.

### Abraham Tull or William Hawkins (Berkshire, 1787)

**Fig. 6:**
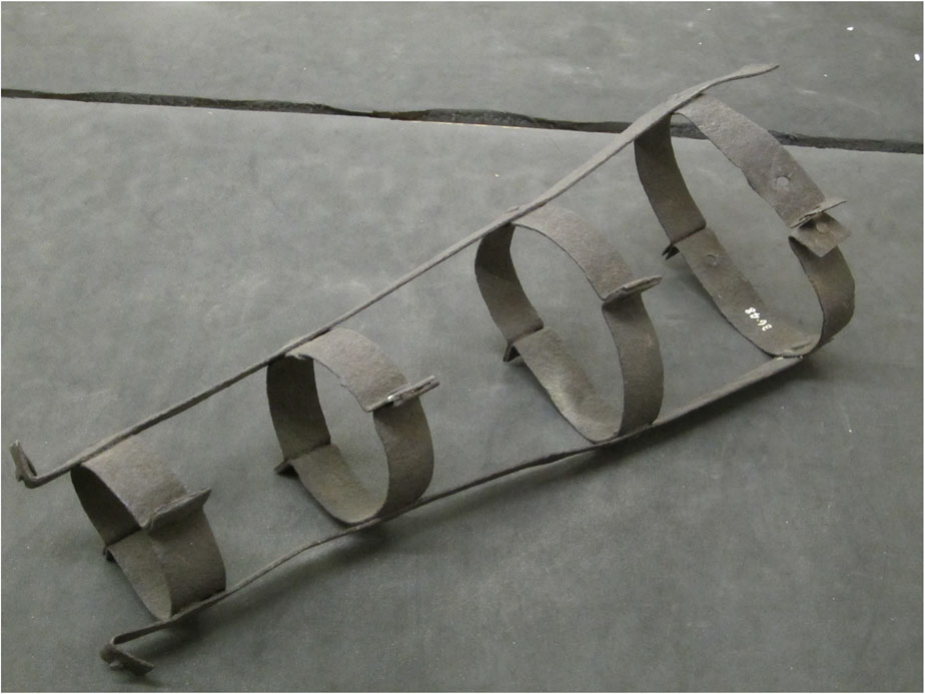
Leg piece of Tull or Hawkins, Reading

Abraham Tull and William Hawkins were executed for the same murder in 1787 and hung in chains on Ufton Common, Berkshire. Their gibbet cages remained in place until a Mrs. Brocas of Beaurepaire “then residing at Whitfield Park” ordered them to be taken down and buried, as the sight of their bodies upset her every time she had to ride past them (Andrews 1899, p. 63). The whereabouts of most of the gibbet cages are unknown, but a single leg piece survives in the collections of Reading Museum (Fig. 6). This takes the form of two straight strips of iron fastened together with iron hoops, each made from two curved and flanged pieces of metal bolted to the inside of the strap. Although the rest of the cage is missing and unrecorded, there is plainly an attachment for some sort of foot piece. The costs of executing Tull and Hawkins and hanging them in chains was more than £60, according to the sheriffs’ cravings.

### Winchester (name and date unknown, possibly Hill 1777)

**Fig. 7:**
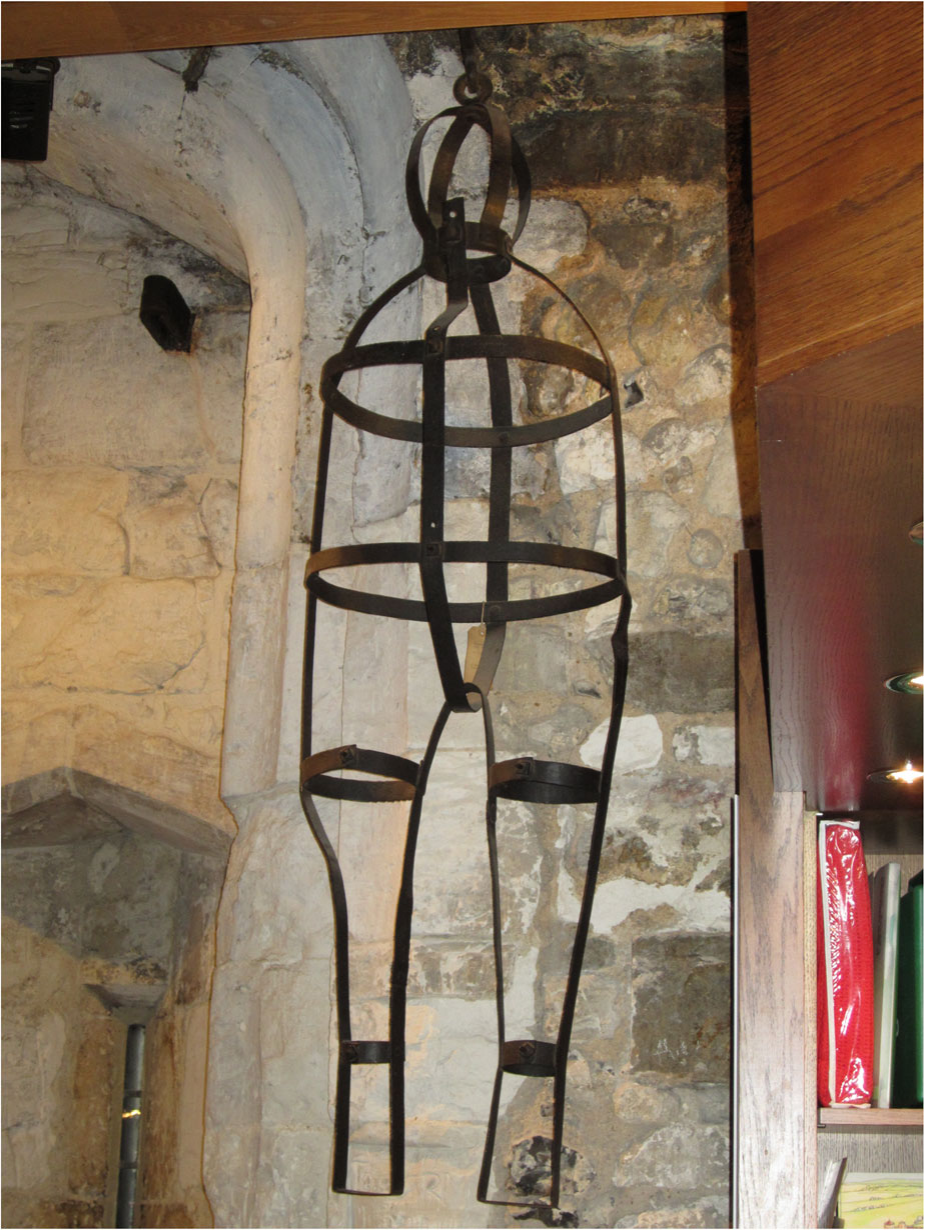
Gibbet cage at Winchester Westgate Museum, possibly that of James Hill

There is a gibbet cage in Winchester Westgate Museum but it is of uncertain provenance (Fig. 7).

Curator Ross Turle writes:

Tradition has it that it was used to display the remains of James Hill (Jack the Painter) who was condemned at Winchester Assizes 1777 for sabotage in Portsmouth dockyards during the American War of Independence, and executed at Blockhouse Point, Portsmouth. We have nothing to confirm this story and an old label read that the cage was from the old Winchester Gaol. This seems more likely as it is recorded that the cage was given to Winchester Museums in 1890 by the H. M. Prison Commissioners, Winchester. By 1890, the prison had moved to the present Romsey Road site but the old gaol had been in Jewry Street (pers. comm.).

Whatever the case, gibbetings did not take place within the jail, so the cage must have come there after use elsewhere; the two stories are not incompatible.

The gibbet has an iron collar from which long strap goes down the torso, between the legs and up the back to attach to the collar. This simple frame has two iron hoops to hold the body in position. The leg pieces are extensions of the vertical sidebars that go down the sides of the body and terminate in stirrups. There are also inner leg straps and two hoops that hold the leg in place. There are no separate arm pieces, but the torso is quite wide and it is possible that the arms were held against the sides of the body.

### Edward Miles (1791)

**Fig. 8:**
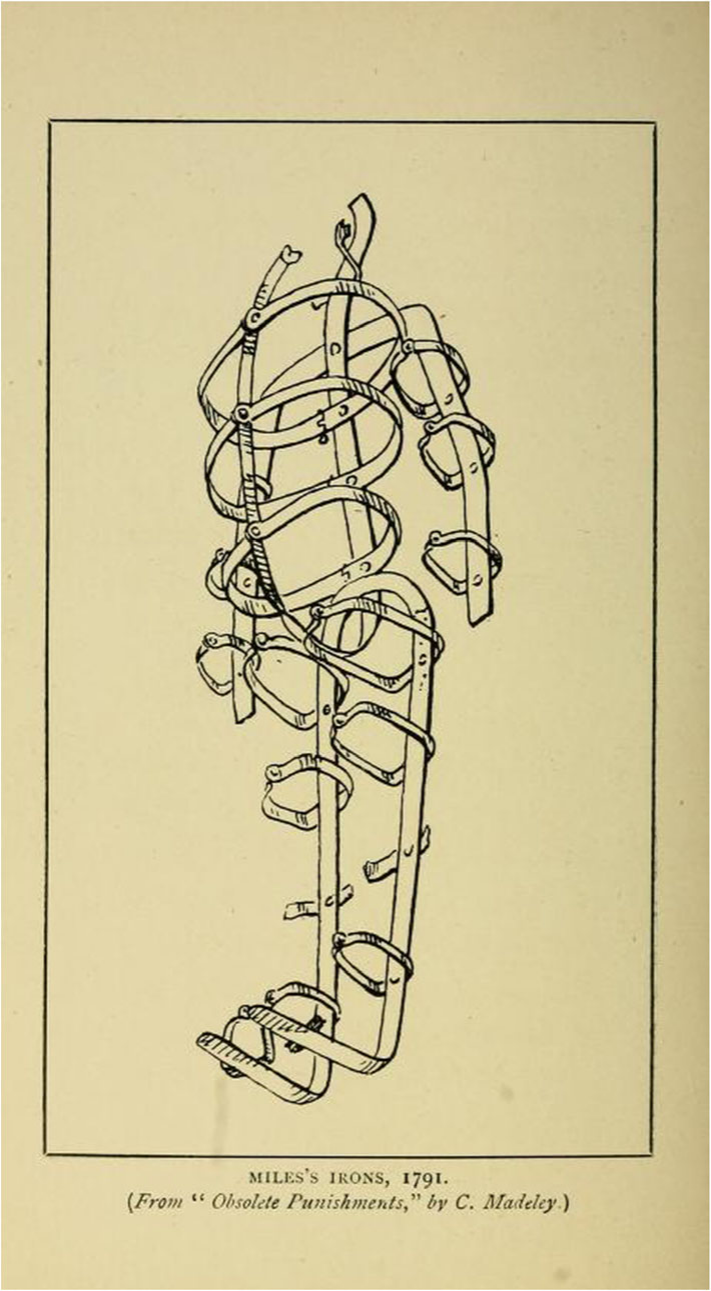
Edward Miles’s gibbet cage, Warrington from Hartshorne 1893

One of the most complete gibbet cages is that in which the body of Edward Miles was displayed after his execution in 1791. Miles’s gibbet cage is now in Warrington Museum and is illustrated in Madeley 1887 (Fig. 8). Iron straps run along the back of the head, the torso, arms and legs, with the two leg straps bent at the ends to form foot supports. To this framework are attached 15 bands to hold the body in place. The headpiece in incomplete and the method of attachment unknown. The straps and bands are not adjustable and so must have been made specifically to fit Miles.

### Spence Broughton (Sheffield, 1792)

Weston Park museum in Sheffield hold an iron belt and a length of chain with something like cuffs on the ends that the catalogue tentatively identifies as belonging to Spence Broughton’s gibbet chains. The belt is fitted with four chain ends and has a hinged latch. The chains have larger rings on the ends that are too large to be manacles, and possibly too large for ankles too. These objects are very different in style to all the other surviving gibbet cages, so if the identification is correct they must have belonged to a very unusual set of chains. Since the identification is based only on the function attributed to the objects when they were donating to the museum in the 1930s by a private individual, there is little else to support this interpretation. The Sheriffs’ Cravings record that the carpenter and smith were paid £9, 15s, 2d for the gibbet and chains—a hefty sum for which one would expect to see something pretty substantial. These objects might be the metal parts of a draught collar for harnessing a heavy horse, and possibly a hame chain, similar to those depicted in Fogg (1981, p. 5), or leg irons for prisoners, similar to those housed in the crime and punishment gallery of Norwich Castle museum.

### John Nicholls (Suffolk, 1794)

**Fig. 9:**
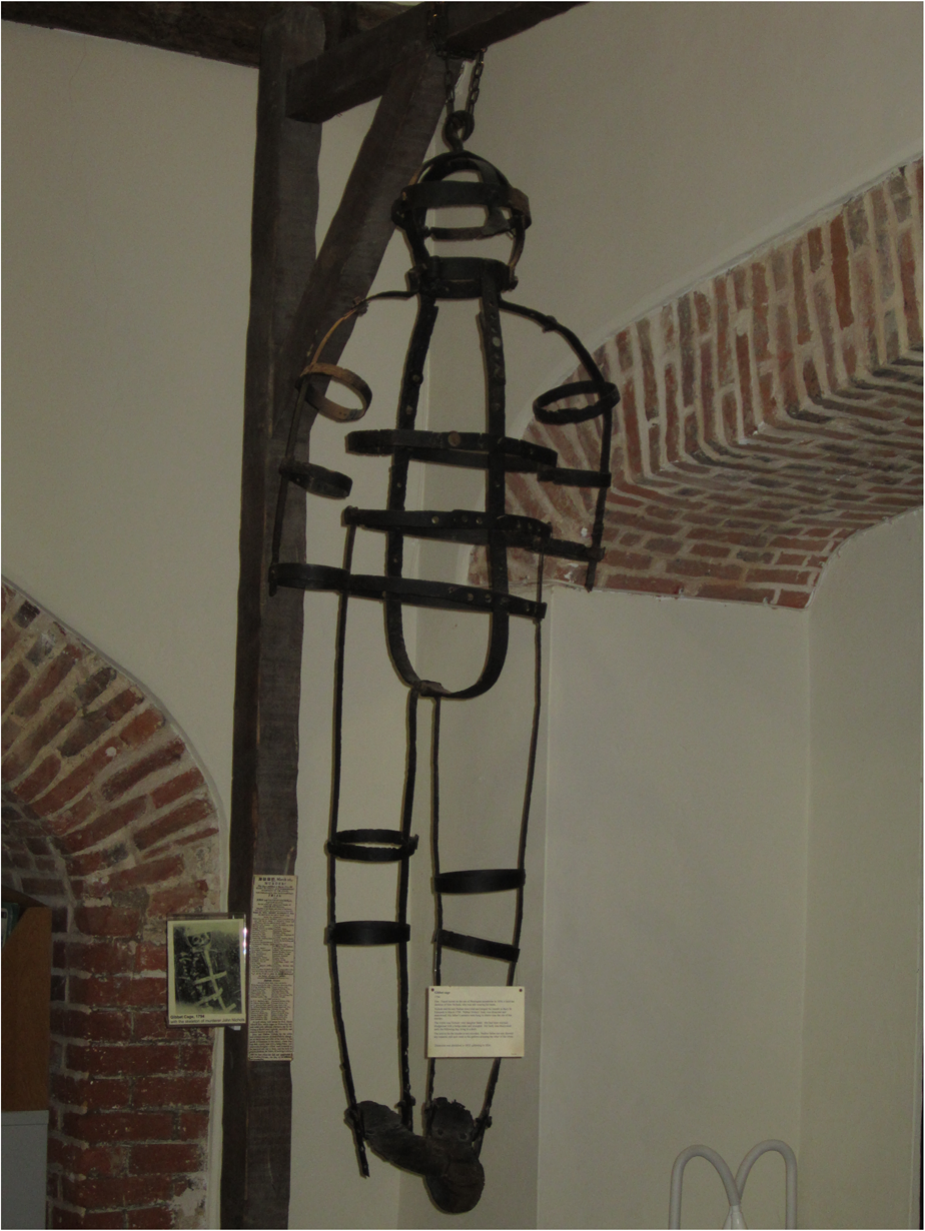
John Nicholls gibbet cage, Bury St Edmunds

This gibbet cage takes the form of a central strap looped between the legs and shaped at the top to form the main element of the headpiece (Fig. 9). This is the only existing gibbet cage with a shaped nosepiece. To the headpiece are attached three hoops that hold the torso, and separate arm and leg elements are connected. These take the form of two long straight bars connected by hoops. Uniquely the arm straps run down the outside of the arms and the hoops are on the inside. The leg pieces have stirrups at the end with two footplates under the feet. The main torso hoops are extensively punched and can be adjusted to fit the girth of the body. This cage is quite similar to Cook’s gibbet, but has fewer hoops along the limbs and is slightly more anatomically shaped. It is on permanent display at Moyse’s Hall museum in Bury St Edmunds, Suffolk. According to the Sherrifs’ Cravings Nicholls’s gibbet irons cost £6, 10s.

### Stephen Watson (Bradenham Heath, Norfolk, 1795)

**Fig. 10:**
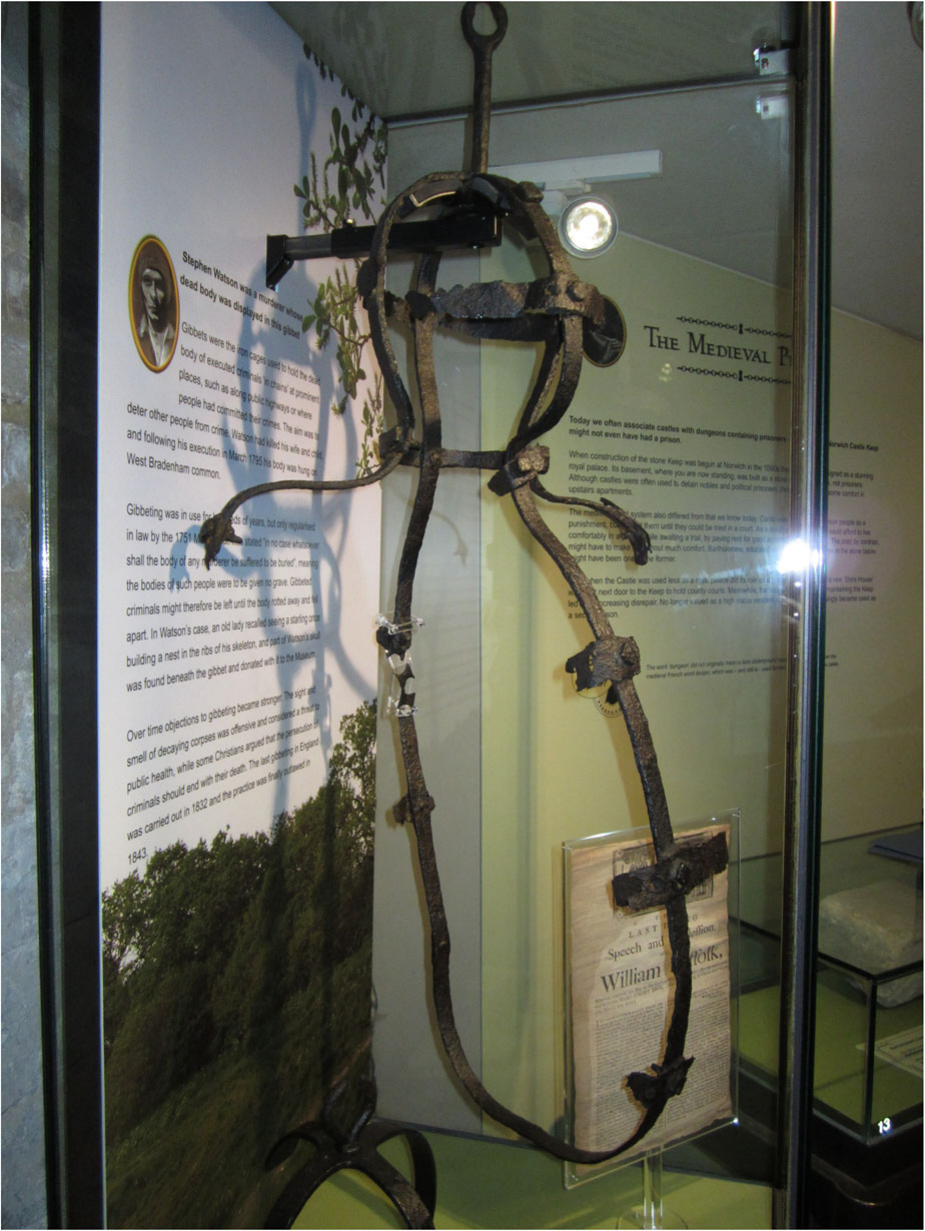
Stephen Watson’s gibbet cage, Norwich

Norwich Castle Museum holds a substantial part of the gibbet cage of Stephen Watson, which was uncovered on Bradenham Common by H. Rider Haggard in 1899. Although affected by rust, the surviving portion of the gibbet comprises a headpiece with four vertical straps, one of which must have covered part of the face (Fig. 10). There is a torso consisting of vertical bars which are continuation of the head piece, and much less substantial horizontal bands which have largely not survived except around the places where they attach to the vertical bars. There is evidence that the horizontal straps were punched several times to be adjustable. No arm or leg pieces survive although a rivet hole in the gusset suggests that leg piece formerly were attached there. The swivel eye shows considerable wear. Associated with this gibbet cage are two skull fragments.

### Thomas Quin or James Culley (Wisbech, Cambridgeshire, 1795)

Wisbech and Fenland Museum hold the headpiece of an iron gibbet relating to either Thomas Quin or James Culley. These were among four Irishmen convicted of murdering William Marriott at Guyhirn, near Wisbech. Two of the murderers were sentenced to dissection, the other two to be hung in chains “opposite the house where the murder was committed,” according to the unpublished Diary of John Peck (1818, p. 134) also kept in the museum. A note added later records: “The gibbet was washed down by a great sea flood coming down the Wash, 1831. For some years before, not a vestige of their bones could be seen. My brother, Joseph Peck of Bevis Hall, has the irons that contained one of the poor men’s skulls in his possession.” The headpiece, known locally as “Paddy’s night cap,” consists of two iron bars joined in the middle and bent around the head to be attached to a hinged collar.

### Thomas Temporell (Tom Otter), (Doddington, Lincolnshire, 1806)

**Fig. 11:**
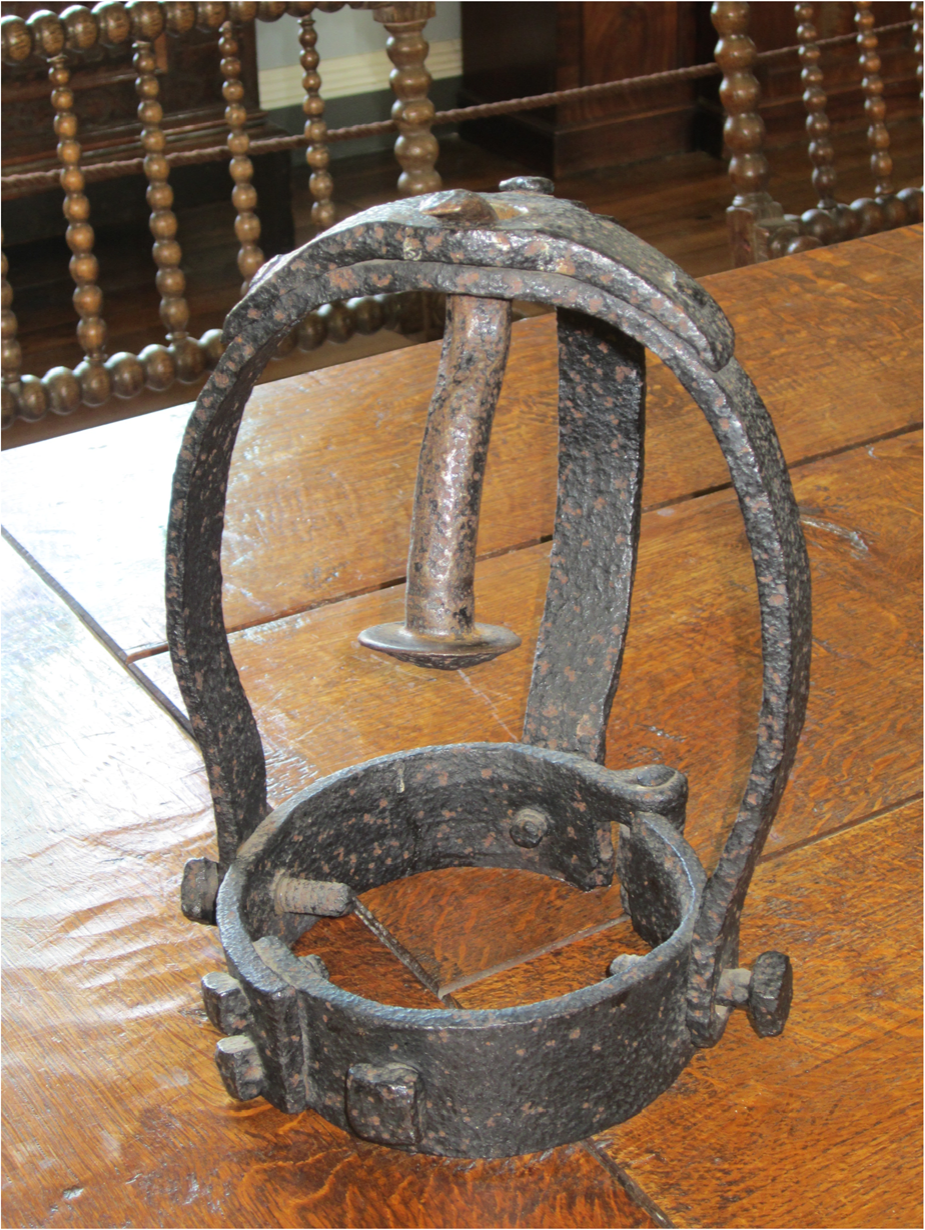
Tom Otter’s gibbet headpiece

Thomas Temporell, known locally as Tom Otter, was hung in chains for the murder of his wife, at Drinsey Nook, near Doddington, Lincolnshire. Only the headpiece and one leg brace now survive and they are in the private collection at Doddington Hall. When Tom Otter’s gibbet was blown down in 1850, 46 years after he was first hung up, the gypsies acted quickly and were able to take nearly all the irons, except for the head piece which was kept by Edwin Jarvis of Doddington Hall who recorded the event in a commonplace book still kept at the hall in the possession of his descendant Claire Birch. The headpiece consists of a very solid and substantial iron piece with three arms bent into a head shape and bolted to a heavy hinged collar (Fig. 11). The top is reinforced with a bolted-on plate and has a central hole through which passes the hook.

### William Jobling (Jarrow, Northumberland, 1832)

**Fig. 12:**
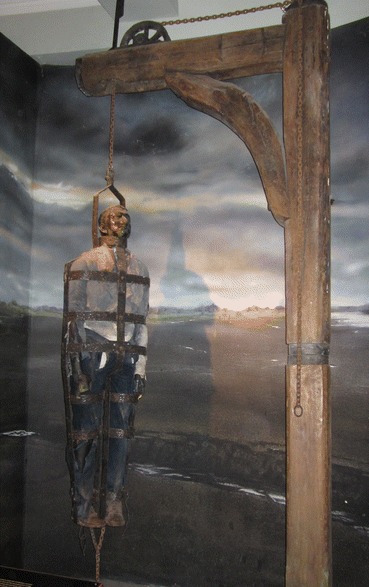
The crossbar and chains of Jobling’s gibbet are original, but the cage is probably a replica

William Jobling and James Cook were the last two men to be hung in chains in England, after the custom had generally fallen into disuse. They were executed within a couple of weeks of one another in August 1832. Given the infrequency of gibbetings by this time, both cases attracted a great deal of attention. Both men were only hung in chains for a short time, however. Jobling’s gibbet was removed by his friends a few weeks after it had been set up in Jarrow Slake, near the scene of the murder in which he was involved. What happened to Jobling’s gibbet after that is rather unclear. Hodgson (1903, p. 377) recounts the story, which also appears in the Proceedings of the Newcastle Society of Antiquaries in 1888, of the rescue of Jobling’s body from Jarrow slake by a group of his friends and relatives. According to this account the gibbet was cut down by sawing through the iron bar which attached it to the crosspiece. Cutting through the iron was so difficult and time-consuming that it was nearly morning by the time they had finished and the body was buried temporarily in the slake (a tidal mud flat in the Tyne estuary) before being removed and buried in a grave dug at the corner of Jarrow Quay the following night. The source of this account—allegedly a deathbed confession by Jobling’s brother-in-law Robert Turner—does not explicitly mention whether the body was still enclosed in its gibbet irons at burial. The account in the Proceedings of the Newcastle Society of Antiquaries—a letter received from Richard Fairlamb of Greatham Hospital, near West Hartlepool retelling what he was told 50 years earlier by a man he worked with who claimed to be one of those who had buried Jobling’s body, mentions explicitly that the corpse was buried “enclosed in the cage, in the south-west corner of Jarrow churchyard.” Certainly, given the (credible) claim that it took all night to cut through one iron bar, the task of cutting his body free of the cage would have been hard to accomplish in the few hours of darkness available to the burial party, and interment within the cage seems most probable. However, the *Proceedings of the Society of Antiquaries of Newcastle* (1888, p. 263) also records the donation in 1888 to the Society by the North Eastern Railway Company of “the ironwork of Jobling’s gibbet from Jarrow Slake.” The subsequent loan of the gibbet to the museum at Jarrow and then to South Shields Museum is well documented, and the South Shields Museum currently has a display of a gibbet post, chains and gibbet cage with a model of Jobling inside (Fig. 12). Whether this is the object that was donated to the Society of Antiquaries, or a replica or a mixture of the two is not clear from the museum’s records. Nor is it clear what exactly was donated to the Society of Antiquaries 140 years ago.

A sketch and costings for a model gibbet cage and mannequin in the museum’s archive and dated to 1999 was never commissioned, according to the model maker (Bob Wakeling, pers. comm.). The one on current display is apparently the same as the cage on display in the 1970s, according to photographs in the museums archive.

**Fig. 13:**
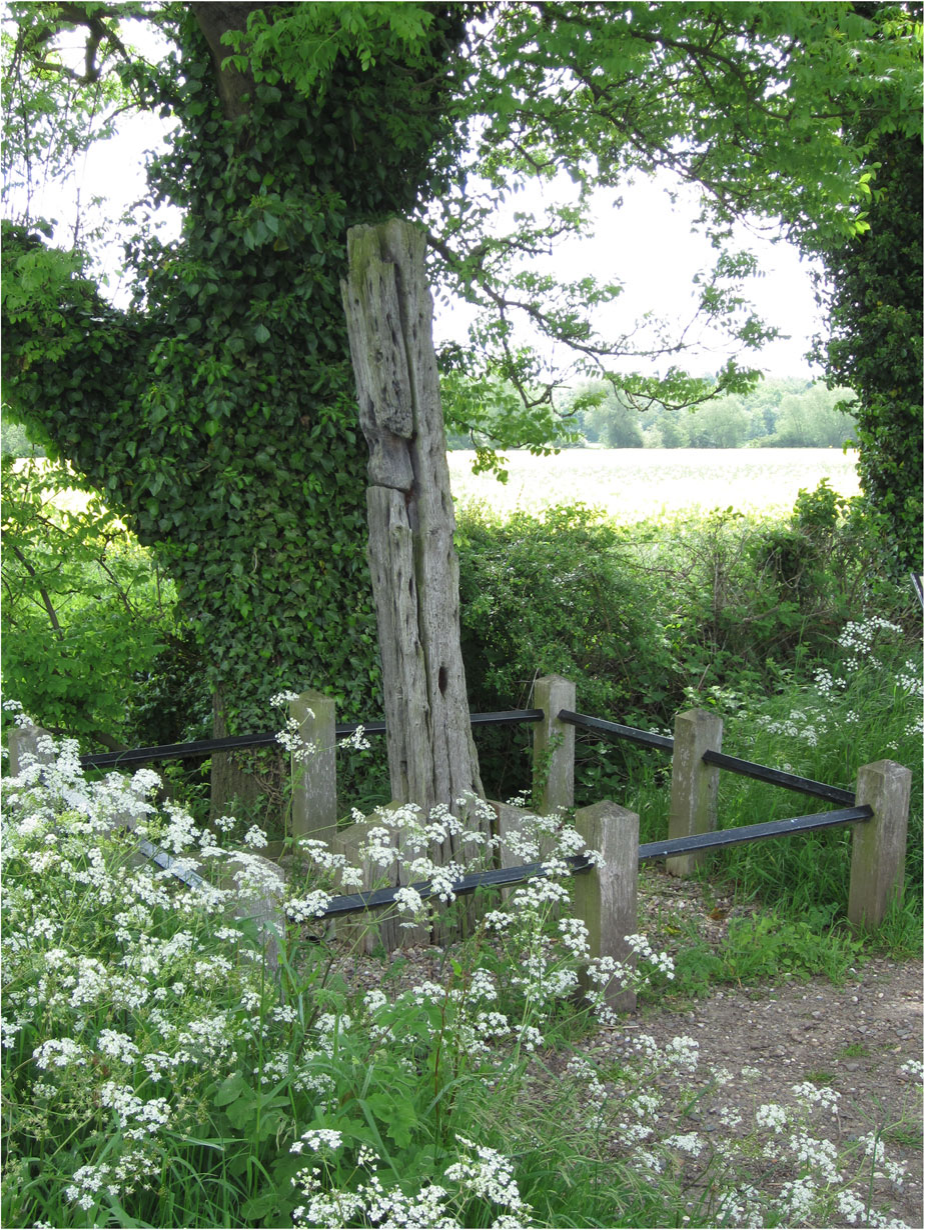
Remains of gibbet post at Congerstone, Leicestershire

Attempts to locate Jobling’s gibbet cage near Jarrow Slake using a mine detector in the early 1970s were unsuccessful (*South Shields Gazette* 1972, p. 10) as were all the extensive attempts to locate Jobling’s burial place by Vincent Rea, local historian, former curator of the Bede gallery at Jarrow and Jobling enthusiast. Newspaper accounts, however, do reveal that the gibbet post remained in the Slake until March 1856 when it was removed by contractors making the Tyne Docks. The gibbet post and pulley-operated chain on display at South Shields is probably original and shows careful workmanship and a degree of ornamentation. The pulley system is elaborate and does not entirely accord with the story of how it took all night to cut through one iron bar—surely the quicker thing to do would have been to cut one of the links to the chain from which the cage was suspended? But it seems an expensive and elaborate structure to make as a replica, and evidently something of the original gibbet, chains and cage was donated to the Antiquaries. I suspect that the rest of the cage is a replica, given the probability that the original was secretly buried in 1832 with Jobling still inside. The post in South Shields museum would thus be a rare surviving example of an upper part of a gibbet post, although there are famous replicas of Winter’s gibbet at Elsdon, Northumberland, Caxton Gibbet, Cambridgeshire and Combe Gibbet, Berkshire (formerly Hampshire). Part of an original gibbet post remains in situ at Congerstone, Leicestershire (Fig. 13); this was erected in 1801 for the body of John Massey (Potter Briscoe 1895, p. 7). What is described in the Oxfordshire Museums catalogue as the crosspiece of Parr’s gibbet, held by Banbury Museum, is far less substantial than Jobling’s, and the identification as the timber from a gibbet (which does not match the text in the gallery) might be apocryphal, or refer to a scaffold for hangings in the town, rathar than the supporting framework for a heavy iron gibbet cage.

### James Cook (Leicestershire, 1832)

James Cook was executed and hung in chains on August 10, 1832, a week after Jobling. He was the last man in England to be so treated. The gibbet cage itself is iron. Straight bars run down the front and back of the torso and along the limbs (there are separate spaces for each arm and each leg) and from these are hinged semi-circles that form a girdle around the body and contain the limbs. The headpiece consists of a thick curved strap that goes up the back of the head and then forms the hook at the top. To this are attached a hoop that passes under the chin and to the top of the head and a horizontal strap across the back of the head. The legs terminate in footplates.

The exhibition of Cook’s body on a pole about 33 ft (10 m) high at the junction of Saffron Lane and Aylstone Road attracted such huge crowds that it was taken down after 3 days by order of the Secretary of State and buried in its cage at the scene of his gibbeting. It was dug up in the twentieth century by workmen carrying out improvements to the junction and the cage was donated to the H. M. Prison Service museum who passed it later to Nottingham’s Galleries of Justice where it remains. A replica is displayed at the Leicester Guildhall.

## The Necessary Functions of a Gibbet

**Fig. 14:**
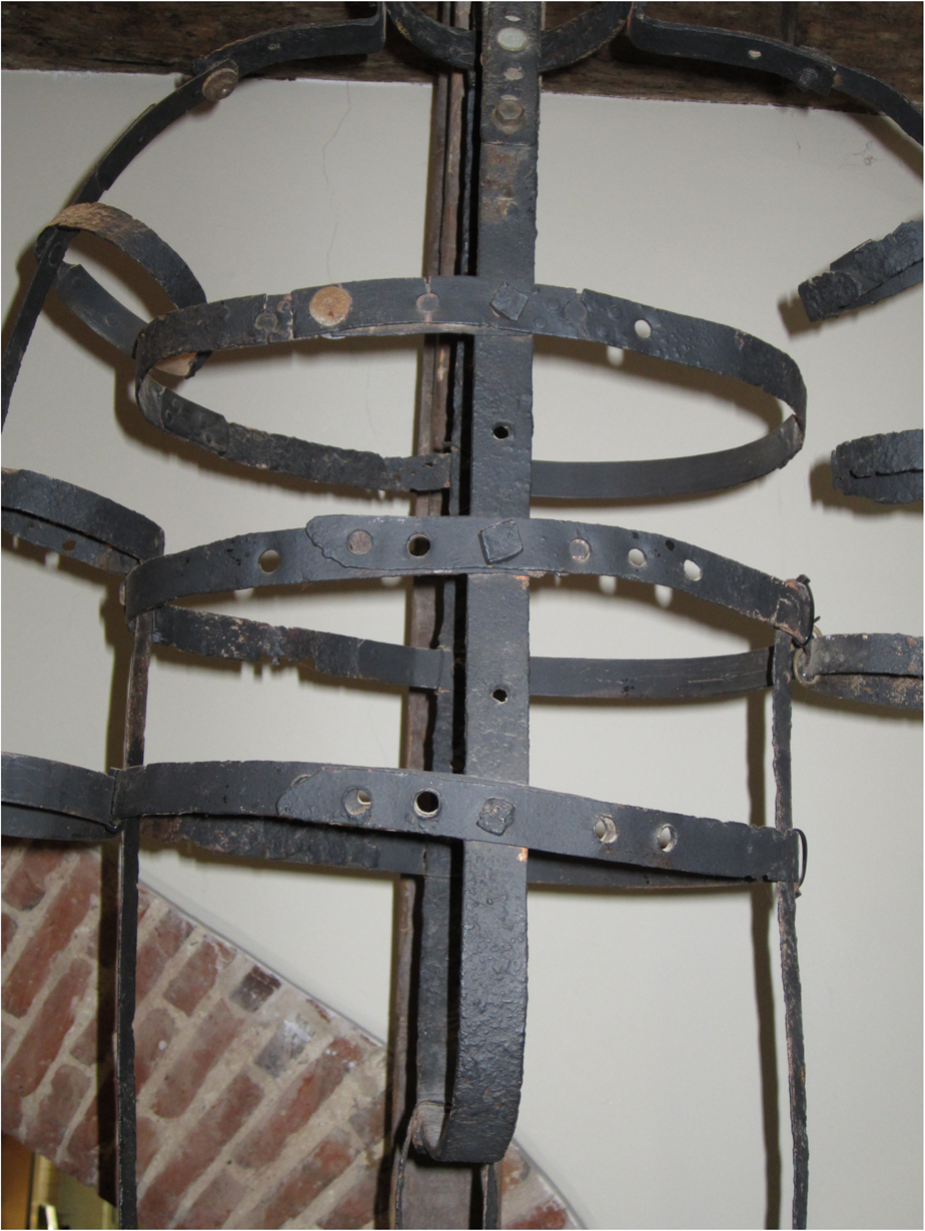
Detail of multiple punched holes on Nicholls’s gibbet so that it could be adjusted for a close fit

What must a gibbet cage do? What functions must an effective set of irons fulfill? First, it must contain the body and prevent it either falling out or being removed, while at the same time still ensuring its visibility. In order to do this, most gibbet cages were designed to fit closely to the body, allowing as much as possible of the body to be visible, while ensuring that the gaps were too small to remove it. When possible the prisoner was measured for his irons before execution, but there were other means of ensuring a close fit, notably construction with punched straps and hoops that could be adjusted to size by riveting (Fig. 14). Bodies in advanced decay will necessarily fall through the framework in pieces, although the skull, if unbroken, might remain in the headpiece, as in the case of John Breeds at Rye or Sion y Gôf at Dylife. In addition, small pieces of the body could easily be removed by animals or birds. However, by adding to the horror of the gibbeted body, such removals did not diminish the power of the spectacle.

Strength and security seem to have been the most valued and discussed features of a good gibbet. The cravings often describe the gibbet as “strong” and sometimes specify the necessity of making theft of the body impossible. The Sheriffs’ Cravings for Bedfordshire in 1738, for example, mention that the irons of John Sturabout cost £7 and 7 shillings “to prevent [his body] being stolen wherein much iron and workmanship is required.” The cravings related to the gibbetings of David Anderson (1736) and William Fairall (1749) in Kent both make explicit reference to the need for security. Anderson’s gibbet was “built in a strong manner and filed with nails and braced with iron to prevent the same from being cut down” and Fairall’s gibbet was also riveted with iron to prevent his fellow smugglers from cutting him down. The journalist for the *Daily Courant* (1731) who reported on John Naden’s hanging in chains in Staffordshire was impressed by the chains, made by somebody from Birmingham “in so curious a Manner, that they will keep his Bones together till they turn to Powder, if the Iron will last so long.”

The measures taken to prevent theft of the body were evidently necessary. Despite the penalty of transportation for those caught removing a body from a gibbet, there are numerous accounts in eighteenth-century newspapers of the theft or attempted theft of bodies from gibbets, usually by friends and relatives seeking to give the man a decent burial. The body of Walter Kidson, also hung in chains in Gloucestershire, on Stourbridge Common in September 1773, was stolen 2 years after his execution. The *London Chronicle* of September 19–21, 1775 (p. 286) reports that the gibbet was sawn off at the neck and the body removed. In 1786, a convict of the Admiralty court, George Coombes, hung in chains at Boar Ness Point, Kent, was stolen, and the Admiralty offered a £50 reward for information leading to the apprehension of those responsible (*London Gazette* 1786, p. 71). Sometimes other motivations also prompted unofficial removal of body parts. Eugene Aram’s skull was taken from his gibbet cage in Knaresborough by a local surgeon who wanted to add it to his private collection (Dobson 1952, p. 272).

Second, the gibbet cage must be conspicuous. Much of this was achieved through the use of a tall pole and advantageous siting (frequently taking advantage of a natural or archaeological eminence such as a hillock or barrow) adjacent to well-used public roads. The successful cage should make the body more visible and more terrible to onlookers. The gibbet cage must contribute to the awe of the spectacle by allowing the body to be seen, and permitting some limited movement. If this also caused the chains to creak, so much the better. W. H. B. Saunders (1888, pp. 103–104) says that the former ostler at an inn in Huntingdonshire recalled seeing Matcham’s gibbet: “It often used to frit me as a lad. I have seen horses frit with it. The coach and carriage people were always on the look out for it. Oh yes! I can remember it rotting away, bit by bit, and the red rags flapping from it. After a while they took it down and very pleased I were to see the last of it.”

Third the gibbet cage had to be durable. The body was supposed to remain up there until it had decayed, and as there was no particular time for taking it down, many gibbets remained in their location for decades. Heavy iron was normally used. The condition of surviving gibbets is testament to their durability, especially since many of them have been hanging outside for many decades, often followed by a period of burial in a wet field.

Fourth, it had to be possible to construct a gibbet cage quickly. As we have seen, in most cases less than a week was available to design and construct the full kit. The smith had to work together with the carpenter whose job it was to make the wooden pole, to ensure that the gibbet was securely erected in time for the arrival of the body, and then to encase the body in its irons and probably to oversee its suspension.

Fifth, while being durable and secure, the gibbet cage also had to be light enough to hoist on a gibbet post which could be around 10 m high, and not so solid that the visibility of the body was in any way impeded. The criminal on the gibbet should be recognizable to those who had known him in life.

## The Technology of the Gibbet

Gibbeting required a pole/scaffold, a length of chain and a gibbet cage or suit. The sheriffs’ cravings normally bundle all these costs together, but sometimes they specify the recipient of the money and the nature of their job. For example, John Bowland’s gibbet, commissioned in Rutland in 1769 cost £5, 15s, 6d for the “set of iron chains,” paid to John Fox, a blacksmith, and £6, 2 s, 6d for the construction and erection of the wooden gibbet frame, paid to John Wyhters, a carpenter.

The gibbet cage is an unusual artfact. It is comparatively rare—only 16 are known, out of only a couple of hundred (at most) that formerly existed in Britain. The infrequency of its manufacture makes it unusual also. Blacksmiths in Britain during the eighteenth and nineteenth centuries generally made objects such as agricultural implements, craft tools, machine and vehicle parts, household objects, and farriery (horse shoeing and horse tack). These artifacts were learned during long apprenticeships and conform to local traditions (Bailey 1977; Collins 1996). By contrast, a gibbet was needed so infrequently that it was not a form within the learned repertoire of most blacksmiths. It is likely that many smiths would never have seen a gibbet cage before they were asked to make one, which had to be completed in only a very few days. Therefore, each blacksmith needed to design a gibbet effectively from scratch. This constant reinvention of the wheel is evident in the proliferation of designs and in the absence of clear typological logic by either region or time period, even though such typologies are observable in other, more frequently made, products of the blacksmith’s craft. The range of designs identified represent independent and idiosyncratic responses to the problem of designing a framework that would enable the range of functions identified above.

## Conclusion

From this extensive review of the practicalities of hanging in chains three things particularly stand out: first, gibbets were rare and expensive. In most parts of the country a gibbeting only occurred very occasionally; most people would only have witnessed one, or none. The construction and erection of the equipment cost as much as a year’s average pay for a laborer. Second, the design, manufacture, and location of gibbets maximized visibility. Tens of thousands of people were afforded a sensory and material encounter with the executed body, both at the time of the gibbeting and during the months and years that followed. Finally, the variability of design and absence of any clear regional or chronological trends demonstrate that the necessary functions of the gibbet were achieved on a local and contingent basis.

The technology of the gibbet is an example of the way that top-down, state-level imperatives found local resolution. During the eighteenth and nineteenth centuries, local traditions in many areas of practice—from manufacture to town planning, burial practice, to childrearing—were increasingly homogenized by their subjection to state regulation. After 1832, there were no geographically variable postmortem punishments: burial of executed criminals was to take place within the prison precinct, and the bodies on the tables of anatomists were those of the poor rather than of malefactors. Gibbeting represents the overlap between statewide homogeneity, enforced by national law, and local expression. The practical details of how the law was to be carried out were left to the discretion of the sheriff and the innovative capacity of the local craftsmen.
